# Effectiveness of Perioperative Cardiopulmonary Rehabilitation in Patients With Lung Cancer Undergoing Video-Assisted Thoracic Surgery

**DOI:** 10.3389/fmed.2022.900165

**Published:** 2022-06-15

**Authors:** Wei-Hao Chao, Sheng-Hui Tuan, En-Kuei Tang, Yi-Ju Tsai, Jing-Hui Chung, Guan-Bo Chen, Ko-Long Lin

**Affiliations:** ^1^Department of Medical Education and Research, Kaohsiung Veterans General Hospital, Kaohsiung City, Taiwan; ^2^Department of Medical Education and Research, Zuoying Branch of Kaohsiung Armed Forces General Hospital, Kaohsiung City, Taiwan; ^3^Institute of Allied Health Sciences, National Cheng Kung University, Tainan City, Taiwan; ^4^Department of Rehabilitation Medicine, Cishan Hospital, Ministry of Health and Welfare, Kaohsiung City, Taiwan; ^5^Department of Surgery, Kaohsiung Veterans General Hospital, Kaohsiung City, Taiwan; ^6^Department of Nursing, Shu-Zen Junior College of Medicine and Management, Kaohsiung City, Taiwan; ^7^Department of Physical Therapy, National Cheng Kung University, Tainan City, Taiwan; ^8^Department of Physical Medicine and Rehabilitation, Kaohsiung Veteran General Hospital, Kaohsiung City, Taiwan; ^9^Department of Internal Medicine, Kaohsiung Armed Forces General Hospital, Kaohsiung City, Taiwan; ^10^School of Medicine, College of Medicine, Kaohsiung Medical University, Kaohsiung City, Taiwan; ^11^School of Medicine, College of Medicine, National Yang Ming Chiao Tung University, Taipei City, Taiwan; ^12^Department of Post-Baccalaureate Medicine, National Sun Yat-Sun University, Kaohsiung City, Taiwan

**Keywords:** lung cancer, perioperative cardiopulmonary rehabilitation, cardiopulmonary exercise testing, video-assisted thoracic surgery, postoperative pulmonary complications

## Abstract

**Objectives:**

Patients with lung cancer pose a high risk of morbidity and mortality after lung resection. Those who receive perioperative cardiopulmonary rehabilitation (PRCR) have better prognosis. Peak oxygen consumption (peak VO_2_), VO_2_ at the ventilatory threshold (VO_2_ at VT), and slope of minute ventilation to carbon dioxide production (V_E_/V_CO2_ slope) measured during pre-surgical cardiopulmonary exercise testing (CPET) have prognostic values after lung resection. We aimed to investigate the influence of individualized PRCR on postoperative complications in patients undergoing video-assisted thoracic surgery (VATS) for lung cancer with different pre-surgical risks.

**Methods:**

This was a retrospective study. We recruited 125 patients who underwent VATS for lung cancer between 2017 and 2021. CPET was administered before surgery to evaluate the risk level and PRCR was performed based on the individual risk level defined by peak VO2, VO2 at VT, and VE/VCO2 slope, respectively. The primary outcomes were intensive care unit (ICU) and hospital lengths of stay, endotracheal intubation time (ETT), and chest tube insertion time (CTT). The secondary outcomes were postoperative complications (PPCs), including subcutaneous emphysema, pneumothorax, pleural effusion, atelectasis, infection, and empyema.

**Results:**

Three intergroup comparisons based on the risk level by peak VO2 (3 groups), VO2 at VT (2 groups), and VE/VCO2 slope (3 groups) were done. All of the comparisons showed no significant differences in both the primary and secondary outcomes (*p* = 0.061–0.910).

**Conclusion:**

Patients with different risk levels showed comparable prognosis and PPCs after undergoing CPET-guided PRCR. PRCR should be encouraged in patients undergoing VATS for lung cancer.

## Introduction

Lung cancer is a leading cause of cancer mortality worldwide in both men and women. In 2020, lung cancer was diagnosed in approximately 2.2 million patients and was responsible for an estimated 1.8 million deaths (1). Lung cancer is categorized into small cell lung cancer and non-small cell lung cancer (NSCLC). NSCLC, which includes adenocarcinoma, squamous cell carcinoma, and large cell carcinoma, accounts for the majority of lung cancer cases. Furthermore, smoking is the predominant risk factor for lung cancer (2). Definitive pathologic results are required to make a diagnosis of lung cancer. Therefore, tissue biopsy is necessary for diagnosis and staging (3). For patients with early disease, surgical biopsy is occasionally preferred because it has the potential to achieve diagnosis and curative resection at the same time. However, minimally invasive procedures are typically preferred in patients with a higher disease stage. In addition, lung resection poses a high risk of morbidity and mortality in patients with severe comorbidities or low cardiopulmonary reserve.

According to the 2016 European Society of Cardiology (ESC) guidelines, cardiopulmonary exercise testing (CPET) can be used to assess perioperative and postoperative risks and has a prognostic value in patients undergoing various surgical procedures, including abdominal aortic aneurysm repair (4, 5), radical cystectomy (6), liver transplantation (7), hepatic resection (8), lung resection (9, 10), bariatric surgery, and colorectal surgery (11). In patients undergoing lung resection, three CPET variables have been proven to have prognostic values: peak oxygen consumption (peak VO_2_) (12), VO_2_ at the ventilatory threshold (VO_2_ at VT), and slope of minute ventilation to carbon dioxide production (V_E_/V_CO2_ slope) (13, 14). Physiotherapy services for patients with lung cancer have historically been hospital based and have focused on postoperative pulmonary complications (PPCs). Although cardiopulmonary rehabilitation is considered an important component of perioperative care in patients undergoing lung resection surgery, previous studies have not investigated the impact of the application of cardiopulmonary rehabilitation before and after surgery (15). Therefore, in this study, we aimed to investigate the influence of individualized perioperative cardiopulmonary rehabilitation on postoperative complications in patients undergoing video-assisted thoracic surgery (VATS) for lung cancer with different pre-surgical risks.

## Methods

### Participants

This was a retrospective study. Patients who underwent VATS for lung cancer at a tertiary medical center (Kaohsiung Veterans General Hospital) between May 7, 2017, and May 3, 2020, were enrolled. Patients with cerebrovascular diseases, severe orthopedic disorders, advanced heart failure (functional class IV), severe valvular diseases, or uncontrolled arrhythmia were excluded. Patients with incomplete medical records or postoperative plain chest radiographs were further excluded. Finally, 125 patients were enrolled for analysis. All patients underwent CPET before VATS. After CPET, the patients underwent perioperative cardiopulmonary rehabilitation based on the determined risk level. All patients received education regarding cardiovascular risk factors from a team comprising doctors, nurses, nutritionists, and physical therapists. This study was approved by the Institutional Review Board of Kaohsiung Veterans General Hospital (number: VGHKS17-CT11-11, date of approval: Oct. 17, 2021).

### Cardiopulmonary Exercise Test

All patients were tested using Metamax 3B (Cortex Biophysik Co., Leipzig, Germany), which consists of a bicycle ergometer, a gas analyzer, and an electrocardiography (ECG) monitor. The patients pedaled on an upright bicycle ergometer for the assessment of peak VO_2_, VO_2_ at VT, and V_E_/V_CO2_ slope. The exercise was started at an intensity of 0-W workload for a 1-min warm-up, followed by incremental loading using a ramp protocol (10 W/min) until exhaustion. The patients were tested with the ramp Bruce protocol, following the guidelines of the American College of Sports Medicine (ACSM). All patients safely completed the test.

### Interventions

The results of CPET were used to classify the risk levels of the patients in accordance with the criteria from the 2016 ESC guidelines (Table 1). All participants were tested for each of the three CPET variables as follows: (1) peak VO_2_, divided into four classes [Weber class A (>20 mL/kg/min), Weber class B (16–20 mL/kg/min), Weber class C (10–15.9 mL/kg/min), and Weber class D (<10 mL/kg/min)]; (2) VO_2_ at VT, divided into two classes [class A (≥11 mL/kg/min) and class B (<11 mL/kg/min)]; and (3) V_E_/V_CO2_ slope, divided into four classes [ventilatory class I (<30), ventilatory class II (30–35.9), ventilatory class III (36–44.9), and ventilatory class IV (>45)](11). Thereafter, all participants were classified into four categories (groups A, B, C, and D) according to their risk level for perioperative or postoperative complications. Group A included patients with Weber class A, VO_2_ at VT class A, ventilatory class I, an increase in systolic blood pressure (SBP) during the exercise test, and ECG results showing no sustained arrhythmias, ectopic foci, or ST-segment changes during the exercise test or recovery phase. Group B included patients with Weber class B and ventilatory class II. Group C included patients with Weber class C, ventilatory class III, flat SBP response during the exercise test, and ECG results showing altered rhythm, ectopic foci, or ST-segment changes during the exercise test or recovery phase without leading to test termination. Group D included patients with Weber class D, VO_2_ at VT class B, ventilatory class IV, decrease in SBP during the exercise test, and ECG results showing altered rhythm, ectopic foci, or ST-segment changes during the exercise test or recovery phase leading to test termination.

**Table 1:** Pre-surgical assessment by cardiopulmonary exercise testing.

**Primary CEPT variables**		
**V_E_/V_CO2_ slope**	**peak V_O2_**	**V_O2_ atVT**
Ventilatory class I V_E_/V_CO2_ slope <30.0	Weber class A peak V_O2_ > 20.0 ml O_2_/kg/min	Class A V_O2_ at VT ≥ 11.0 ml O_2_/kg/min
Ventilatory class II V_E_/V_CO2_ slope 30.0–35.9	Weber class B peak V_O2_ 16.0–20.0 ml O_2_/kg/min	
Ventilatory class III V_E_/V_CO2_ slope 36.0–44.9	Weber class C peak V_O2_ 10.0–15.9 ml O_2_/kg/min	Class B V_O2_ at VT <11.0 ml O_2_/kg/min
Ventilatory class IV V_E_/V_CO2_ slope ≥ 45.0	Weber class D peak V_O2_ <10.0 ml O_2_/kg/min	
**Standard ET variables**	
**Hemodynamics**	**ECG**
Rise in systolic BP during ET (Group A)	No sustained arrhythmias, ectopic foci, or ST-segment changes during ET or in recovery (Group A)
Flat systolic BP response during ET (Group A)	Altered rhythm, ectopic foci, or ST-segment changes during ET or in recovery; did not lead to test termination (Group A)
Drop in systolic BP during ET (Group D)	Altered rhythm, ectopic foci, or ST-segment changes during ET or in recovery; lead to test termination (Group D)
**Patient reason for test termination**	
**Lower-extremity muscle fatigue**	**Angina or dyspnea**
**Chart interpretation**	
Variables in Group A	Excellent prognosis and low risk for perisurgical/ postsurgical complications.
Variables in Group B	Progressively worse prognosis and higher risk for perisurgical/ postsurgical complications.
Variables in Group C	
Variables in Group D	Risk for major adverse event or perisurgical/ postsurgical complications is extremely high; long-term prognosis is poor.

BP, blood pressure; ET, exercise test; V_E_/V_CO2_, minute ventilation/carbon dioxide production; V_O2_, oxygen consumption. ^*^Peak V_O2_ valid if peak respiratory exchange ratio is at least 1.00 or test is terminated secondary to abnormal hemodynamic or ECG exercise response.

### Preoperative Phase

**Figure 1:**
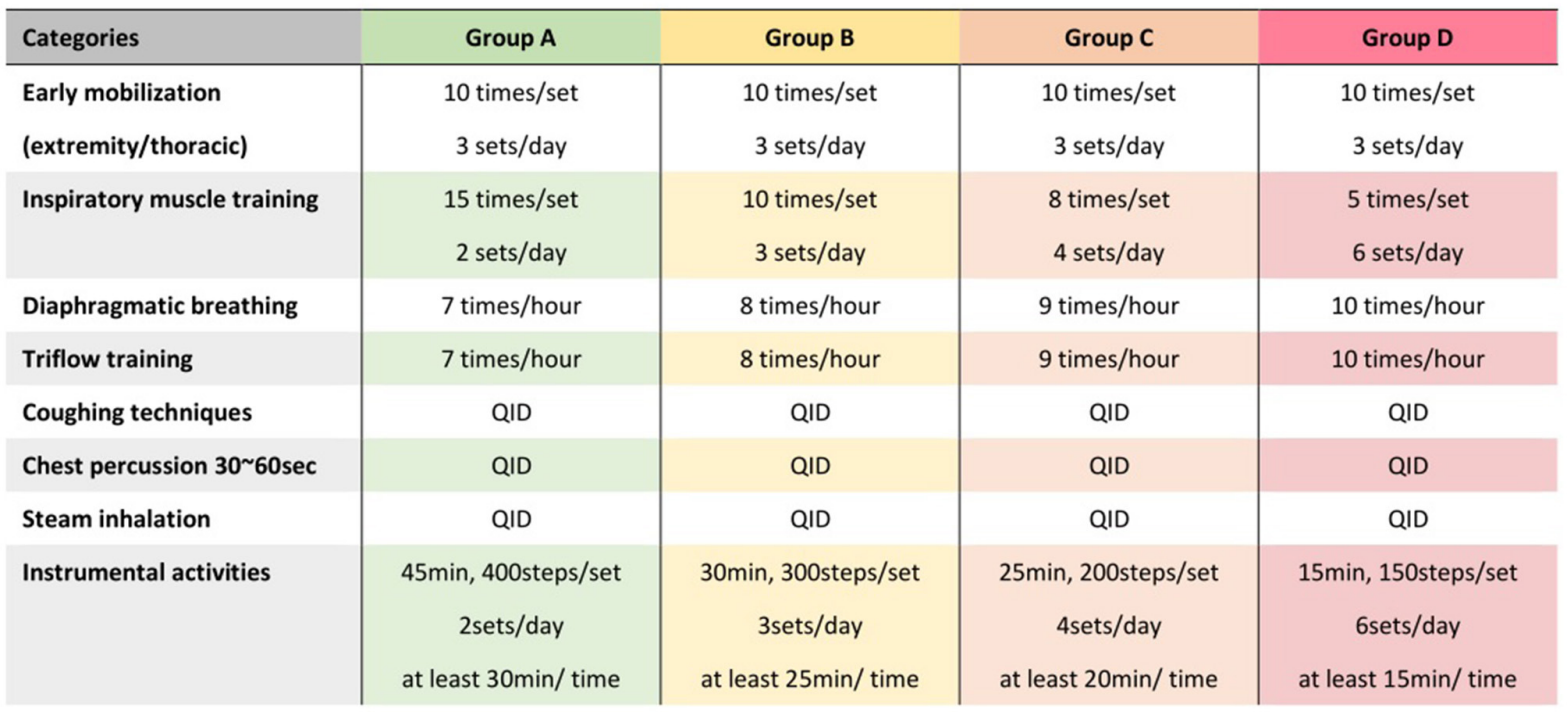
Protocol of perioperative cardiopulmonary rehabilitation for patients with different risk level. Different protocols of perioperative cardiopulmonary rehabilitation were designed for risk levels from group A to group D, respectively, based on the 2016 European Society of Cardiology guidelines (Table 1).

In the preoperative phase, one experienced physical therapist (Jing-Hui Chung) explained the treatment plan and educated the patients regarding common PPCs before starting the training program. All patients were treated with perioperative cardiopulmonary rehabilitation. The perioperative cardiopulmonary rehabilitation used in our study consisted of deep breathing exercises, coughing techniques, early mobilization, and progressive shoulder/thoracic mobility exercises. We used different frequencies and intensities of perioperative cardiopulmonary rehabilitation according to the risk level of each patient before surgery (Figure 1). For example, participants in group B underwent perioperative cardiopulmonary rehabilitation consisting of early extremity and thoracic mobilization, diaphragmatic breathing, Triflow training, and inspiratory muscle training (IMT). The Triflow training was done by a Tri-ball incentive spirometer (Galemed, Taipei, Taiwan) designed to aid in maintaining the lung capacity by encouraging deep and slow breathing. The IMT training was done by using a threshold-type breathing trainer (Dofin DT11/14, Galemed, Taipei, Taiwan). Each IMT session consisted of three sets of 10 breaths with a 2-min break between each set at a target intensity of 30% of the maximal inspiratory pressure (MIP). The MIP were measured using a digital pressure gauge (GB60, Jitto International, Taipei, Taiwan).

### Postoperative Phase

In the postoperative phase, the physiotherapy exercises performed on the first postoperative day included early mobilization, sitting out of bed, stepping and walking in the ward, breathing exercise, incentive spirometry, chest physical therapy, and supported coughing. On postoperative days 1 to 3, the levels of activities were progressively increased (e.g., walking for 5–10 min in the hallway two to three times per day on the first day and then three to four times per day on the next day). The first walking session was supervised by a physical therapist. Deep breathing exercise, thoracic expansion exercise, shoulder/thoracic stretch, and range-of-motion exercise were conducted to improve functional ability in the short term. For example, after surgery, we provided the patients with an IMT protocol in which each session consisted of two sets of 30 breaths with a 2-min break between each set at a target intensity of 15% of the maximal inspiratory pressure, which was incrementally increased by 2 cm H_2_O per day depending on the patients' ability. The patients underwent daily progressive strength and endurance training with aerobic exercise, breathing exercise for lung expansion, and chest physiotherapy intervention before discharge.

### Post-surgical Pain Management

All the recruited patients received acute post-surgical pain management by multimodal approach, which consists in the concomitant use of different analgesic drugs to guarantee the greatest pain relief together with opioid-sparing effect (16). The patients received systemic analgesia, including acetaminophen, non-steroidal anti-inflammatory drugs (NSAIDs), and opioids (from weak opioid, such as tramadol and codeine, to morphine). If the systemic analgesia failed, additional local regional analgesia *via* thoracic paravertebral block (TPB) would be done by anaesthesiologists after evaluation is provided and reviewed by the team and regional analgesia (17). The analgesic management ceased once the chest tube was removed.

### Outcomes Measured

To evaluate surgical prognosis and PPCs, we collected data on several variables assessed during hospitalization. The primary outcomes of this study were variables related to prognosis, including intensive care unit (ICU) length of stay, hospital length of stay, endotracheal intubation time (ETT), and chest tube insertion time (CTT). ICU length of stay was defined as the duration between the surgery end time according to the operation record and the precise time of transporting the patient to the general ward according to the treatment order, calculated in minutes. Hospital length of stay was defined as the interval between the admission time and discharge time, calculated in days. ETT was defined as the duration between the surgery starting time according to the operation record and the precise time of endotracheal tube removal according to the treatment order. CTT was defined as the duration between the surgery end time according to the operation record and the precise time of chest tube removal according to the treatment order.

The secondary outcomes were postoperative complications determined by clinical assessment or imaging evaluations, including subcutaneous emphysema, pneumothorax, pleural effusion, atelectasis, infection, and empyema. To assess postoperative complications, two experienced doctors (Wei-Hao Chao and Ko-Long Lin) investigated all hospitalization records, including progression notes, discharge notes, and nursing records, for detailed physical examination data during hospitalization, especially during the postoperative period. For imaging-diagnosed postoperative complications, one doctor (Wei-Hao Chao) investigated the formal reports of preoperative and postoperative plain chest radiography (CXR) evaluations, which were conducted by an expert radiologic technologist and were interpreted by a radiologist. Another doctor (Ko-Long Lin) confirmed the plain imaging findings on a picture archiving and communication system workstation. We compared the preoperative and postoperative images to identify differences in imaging findings. Finally, clinical complications were divided into four aspects: air (subcutaneous emphysema, pneumothorax, and continuous air leakage observed in a water-seal bottle), fluid (pleural effusion), lung (atelectasis), and infection (fever and empyema). CXR-diagnosed complications were also divided into four aspects: air (subcutaneous emphysema, pneumothorax, pneumomediastinum, and hydropneumothorax), fluid (pleural effusion and pulmonary edema), lung (atelectasis), and infection (pneumonia and empyema). Each aspect accounted for 1 point. Clinical or CXR-diagnosed complications were divided into four aspects: air, fluid, lung, and infection. Each aspect accounted for 1 point. The patients were assessed for either clinical complications, CXR-diagnosed complications, or both.

### Statistical Analysis

SPSS for Windows (version 19.0, released in 2010; IBM Corp., Armonk, NY, USA) was used for all analyses. Continuous variables were expressed as mean ± standard deviation, and categorical variables were presented as absolute numbers and percentages. Data were tested for normality and homoscedasticity before each analysis. The independent *t* test was used to compare outcomes between two different groups. For outcome comparisons among three or more groups, we used one-way analysis of variance, except for gender, type of lung resection, and use of post-surgical pain management, which were done by Chi square test. Moreover, given that there were more than 20% cells have expected frequency <5 when comparing the difference of type of lung resection between different groups based on the classification by ESC, we collapsed the number of lobectomy and pneumonectomy into one row. Statistical significance was set at *p* < 0.05.

## Results

### Basic Characteristics

Patients who underwent VATS for lung cancer during the inclusion period were identified. Patients with cerebrovascular diseases, severe orthopedic disorders, advanced heart failure (functional class IV), severe valvular diseases, or uncontrolled arrhythmia were excluded. Those with incomplete medical records and plain radiographs were also excluded. Finally, 125 patients were included for analysis. Among the 125 participants, 74 (59.2%) were men and 51 (40.8%) were women. The average age, height, weight, and body mass index (BMI) were 59.88 ± 9.61 years, 161.00 ± 8.32 cm, 62.86 ± 11.02 kg, and 24.16 ± 3.22 kg/m^2^, respectively. In terms of the type of lung cancer, 7 (5.6%), 86 (68.8%), and 32 (25.6%) patients had squamous cell carcinoma, adenocarcinoma, and other types, respectively. In terms of types of VATS, 65 (52.0%), 56 (44.8%), and 4 (3.2%) patients received lobectomy, wedge resection, and pneumonectomy, respectively.

The baseline characteristics of each group defined by peak VO_2_ are shown in Table 2A. We found significant differences in age, body weight, BMI, body fat, resting SBP, forced vital capacity (FVC), forced expiratory volume in 1 second (FEV1), and maximum voluntary ventilation (MVV). Patients with lower peak VO_2_ had older age; higher body weight, BMI, body fat, and resting SBP; and lower FVC, FEV1, and MVV. The baseline characteristics of each group defined by VO_2_ at VT are shown in Table 2B. Significant differences were found in age, body weight, BMI, FVC/predicted FVC, FEV1/predicted FEV1, and MVV/predicted MVV. Patients with lower VO_2_ at VT had older age, higher body weight and BMI, and lower FVC/predicted FVC, FEV1/predicted FEV1, and MVV/predicted MVV. The baseline characteristics of each group defined by V_E_/V_CO2_ slope are shown in Table 2C. Significant differences were found in age, FEV1, and MVV. Patients with higher V_E_/V_CO2_ slope had older age, lower FEV1, and lower MVV. In addition, no significant difference of the type of lung resection was noted between each group defined by peak VO_2_ (*p* = 0.896), VO_2_ at VT (*p* = 0.821), and V_E_/V_CO2_ slope (*p* = 0.894).

**Table 2A:** Baseline characteristics of each group defined by peak oxygen consumption (peak VO_2_).

	**Weber class A** **(*n* = 38)**	**Weber class B** **(*n* = 47)**	**Weber class C** **(*n* = 39)**	***P*-value**
Gender (M/F)	24/14	28/19	21/18	0.703
Age (years old)	55.05 ± 9.03	59.09 ± 8.67	65.18 ± 8.53	<0.001*^, *a, b*^
Body weight (Kg)	59.09 ± 8.43	63.51 ± 11.24	65.64 ± 12.29	0.028*^, *a*^
Height (cm)	161.10 ± 7.84	162.35 ± 7.88	159.34 ± 9.24	0.251
BMI	22.72 ± 2.35	23.95 ± 2.95	25.75 ± 3.61	<0.001*^, *a, b*^
Body fat (%)	25.07 ± 6.42	29.37 ± 4.90	30.55 ± 6.78	<0.001*^, *a, c*^
Resting SBP	122.42 ± 17.74	126.61 ± 18.08	134.03 ± 23.13	0.035*^, *a*^
Resting DBP	71.82 ± 9.26	75.17 ± 9.57	74.38 ± 13.28	0.347
Resting HR	78.16 ± 11.41	78.85 ± 12.96	74.38 ± 11.76	0.207
FVC	3.02 ± 0.82	2.74 ± 0.74	2.43 ± 0.70	0.004*^, *a*^
FVC/predicted FVC (%)	103.08 ± 17.32	97.86 ± 18.72	93.75 ± 20.00	0.095
FEV1	2.48 ± 0.67	2.27 ± 0.57	1.96 ± 0.57	0.001*^, *a*^
FEV1/predicted FEV1 (%)	103.51 ± 21.31	99.31 ± 17.52	94.70 ± 22.69	0.171
FEV1/FVC	82.05 ± 7.61	83.03 ± 5.31	80.92 ± 8.32	0.444
MVV	81.16 ± 26.21	77.63 ± 22.92	61.87 ± 22.13	0.001*^, *a, c*^
MVV/predicted MVV (%)	82.38 ± 20.58	84.89 ± 17.03	71.96 ± 17.58	0.006*^, *a, c*^
MIP	−96.33 ± 37.09	−100.65 ± 37.61	−94.71 ± 38.54	0.193
Wedge resection/lobectomy/ pneumonectomy (No.)	18/19/1	20/26/1	18/19/2	0.896

Please refer to Table 1 for the Weber classification. M/F, male/ female; BMI, body mass index; SBP, systolic blood pressure; DBP, diastolic blood pressure; HR, heart rate; FVC, forced vital capacity; FEV1, forced expiratory volume in 1 s; MVV, maximum voluntary ventilation; MIP, maximum inspiratory pressure. *: p <0.05. ^a^: post-hoc analysis by Bonferroni test found significant difference between class C and class A. ^b^: post-hoc analysis by Bonferroni test found significant difference between class C and class B. ^c^: post-hoc analysis by Bonferroni test found significant difference between class B and class A. All the comparisons between different groups were done by one-way ANOVA except for gender and type of lung resection, which was done by Chi square test.

**Table 2B:** Baseline characteristics of each group defined by oxygen consumption at anaerobic threshold (VO_2_ at VT).

	**Class A (*n* = 79)**	**Class B (*n* = 46)**	***P*-value**
Gender (M/F)	45/34	29/17	0.505
Age (years old)	57.80 ± 9.57	63.46 ± 8.65	<0.01*
Body weight (Kg)	59.65 ± 8.65	69.38 ± 12.49	<0.001*
Height (cm)	160.27 ± 7.44	162.25 ± 9.60	0.202
BMI	23.17 ± 2.56	25.87 ± 3.54	<0.001*
Body fat (%)	27.77 ± 6.27	29.54 ± 6.35	0.132
SBP rest	124.96 ± 17.92	132.15 ± 22.67	0.053
DBP rest	73.28 ± 9.41	74.83 ± 12.81	0.479
HR rest	78.33 ± 12.09	75.72 ± 12.43	0.252
FVC	2.78 ± 0.78	2.62 ± 0.77	0.247
FVC/predicted FVC (%)	101.01 ± 18.61	92.54 ± 18.95	0.016*
FEV1	2.30 ± 0.62	2.11 ± 0.63	0.108
FEV1/predicted FEV1 (%)	102.11 ± 20.09	93.23 ± 20.91	0.021*
FEV1/FVC	82.58 ± 7.46	80.97 ± 7.88	0.256
MVV	76.71 ± 23.46	68.70 ± 26.91	0.094
MVV/predicted MVV (%)	83.51 ± 18.66	73.98 ± 18.46	0.009*
MIP	−96.80 ± 34.06	−86.18 ± 54.45	0.548
Wedge resection/lobectomy/ pneumonectomy (No.)	36/41/2	20/24/2	0.821

Please refer to Table 1 for the definitions of class A and B. M/F, male/ female; BMI, body mass index; SBP, systolic blood pressure; DBP, diastolic blood pressure; HR, heart rate; FVC, forced vital capacity; FEV1, forced expiratory volume in 1 second; MVV, maximum voluntary ventilation; MIP, maximum inspiratory pressure. All the comparisons between different groups were done by one-way ANOVA except for gender and type of lung resection, which was done by Chi square test. *p <0.05.

**Table 2C:** Baseline characteristics of each group defined by slope of minute ventilation and carbon dioxide production (V_E_/V_CO2_ slope).

	**Ventilatory class I** **(*n* = 90)**	**Ventilatory class II** **(*n* = 29)**	**Ventilatory class III** **(*n* = 6)**	***P*-value**
Gender (M/F)	57/33	14/15	3/3	0.320
Age (years old)	57.87 ± 9.49	64.48 ± 7.74	67.83 ± 8.93	<0.001*^, *a, b*^
Body weight (Kg)	62.68 ± 10.67	63.77 ± 12.96	61.30 ± 6.10	0.845
Height (cm)	161.95 ± 8.12	158.54 ± 8.04	158.67 ± 11.08	0.124
BMI	23.78 ± 2.76	25.27 ± 4.29	24.50 ± 3.05	0.095
Body fat (%)	28.00 ± 5.76	29.40 ± 7.09	29.97 ± 10.51	0.489
SBP rest	125.74 ± 16.82	133.48 ± 28.34	127.33 ± 11.69	0.196
DBP rest	73.87 ± 10.68	74.72 ± 11.86	69.50 ± 5.58	0.561
HR rest	78.25 ± 11.50	75.97 ± 13.74	71.00 ± 14.89	0.294
FVC	2.82 ± 0.77	2.50 ± 0.72	2.30 ± 0.94	0.063
FVC/predicted FVC (%)	98.50 ± 17.33	97.61 ± 24.03	90.12 ± 19.92	0.583
FEV1	2.34 ± 0.63	1.98 ± 0.54	1.81 ± 0.73	0.007*^, *a*^
FEV1/predicted FEV1 (%)	100.22 ± 19.34	96.58 ± 24.42	89.02 ± 22.72	0.356
FEV1/FVC	82.94 ± 7.42	79.69 ± 8.05	78.78 ± 6.40	0.077
MVV	77.48 ± 26.06	64.63 ± 18.88	62.92 ± 22.71	0.035*
MVV/predicted MVV (%)	82.25 ± 18.87	74.66 ± 19.25	72.63 ± 17.25	0.123
MIP	−96.99 ± 37.17	−90.61 ± 43.41	−88.29 ± 35.26	0.321
Wedge resection/lobectomy/ pneumonectomy (*N*)	41/45/4	12/17/0	3/3/0	0.894

Please refer to Table 1 for the definitions of class I, II, and III. M/F, male/ female; BMI, body mass index; SBP, systolic blood pressure; DBP, diastolic blood pressure; HR, heart rate; FVC, forced vital capacity; FEV1, forced expiratory volume in 1 s; MVV, maximum voluntary ventilation; MIP, maximum inspiratory pressure. *: p <0.05. ^a^: post-hoc analysis by Bonferroni test found significant difference between class I and class II. ^b^: post-hoc analysis by Bonferroni test found significant difference between class I and class III. All the comparisons between different groups were done by one-way ANOVA except for gender and type of lung resection, which was done by Chi square test.

### Comparisons of Outcomes According to Peak VO_2_

On the basis of the 2016 ESC guidelines, 38 patients were in class A (peak VO_2_ > 20 mL/kg/min), 47 patients were in class B (16 < peak VO_2_ <20 mL/kg/min), 39 patients were in class C (10 < peak VO_2_ <15.9 mL/kg/min), and one patient was in class D (peak VO_2_ <10 mL/kg/min). Patients in class D were excluded from outcome comparisons owing to their small number. As for post-surgical pain management, 9, 10, and 11 patients received acetaminophen in class A, class B, and class C, respectively (*p* = 0.306). All the patients in each class received NSAIDs (regular use) and opioids (use on requirement). The mean number of using of different types of NSAIDs and opioids was 0.97 ± 0.28 and 1.39 ± 0.75 in class A, 1.02 ± 0.15 and 1.81 ± 0.92 in class B, and 1.03 ± 0.43 and 1.79 ± 0.95 class C, respectively (*p* = 0.868 and 0.132, respectively). One patient in class B and one patient in class C received additional local regional analgesia *via* TPB.

The outcome comparisons according to peak VO_2_ are shown in Table 3A. In terms of the primary outcomes, the three different peak VO_2_ classes showed no significant differences in ICU length of stay (*p* = 0.061), hospital length of stay (*p* = 0.608), ETT (*p* = 0.189), and CTT (*p* = 0.616). In terms of the secondary outcomes, no significant differences were observed in clinical complications (*p* = 0.363), CXR-diagnosed complications (*p* = 0.321), and clinical or CXR-diagnosed complications (*p* = 0.210).

**Table 3A:** Outcomes comparisons by peak oxygen consumption (peak VO_2_).

			***P*-value**
**Primary outcomes**			
ICU length of stay (hour)	class A (*n* = 38)	6.93	0.061
	class B (*n* = 47)	9.6046	
	class C (*n* = 39)	10.43	
Hospital length of stay (day)	class A (*n =* 38)	8.03	0.608
	class B (*n =* 47)	8.40	
	class C (*n =* 39)	8.18	
Endotracheal intubation time (hour)	class A (*n =* 38)	6.86	0.189
	class B (*n =* 47)	10.41	
	class C (*n =* 39)	11.23	
Chest tube insertion time (hour)	class A (*n =* 37)	103.63	0.616
	class B (*n =* 47)	112.19	
	class C (*n =* 39)	108.63	
**Secondary outcomes**			
Clinical complication	class A (*n =* 38)	0.47	0.363
	class B (*n =* 47)	0.57	
	class C (*n =* 39)	0.72	
CXR- diagnosed complication	class A (*n =* 38)	1.00	0.321
	class B (*n =* 47)	1.06	
	class C (*n =* 39)	1.31	
Clinical or CXR- diagnosed complication	class A (*n =* 38)	1.42	0.210
	class B (*n =* 47)	1.60	
	class C (*n =* 39)	1.95	

*Clinical complications included 4 aspects: air (subcutaneous emphysema, pneumothorax, continuous air leakage over water-seal bottle), fluid (pleural effusion), lung (atelectasis), infection (fever, empyema); each aspect counted for 1 point. *CXR-diagnosed complication included 4 aspects: air (subcutaneous emphysema, pneumothorax, pneumomediastinum, hydropneumothorax), fluid (pleural effusion, pulmonary edema), lung (atelectasis), infection (pneumonia, empyema); each aspect counted for 1 point.

*Clinical or CXR-diagnosed complication included 4 aspects: air, fluid, lung, infection; each aspect counted for 1 point either clinical complication, CXR- diagnosed complication or both were noted.

*One participant was excluded from chest tube insertion time due to dischargement without removing chest tube.

*Weber class D was excluded due to insufficient samples (Weber class D, n = 1).

### Comparisons of Outcomes According to VO_2_ at VT

On the basis of the 2016 ESC guidelines, 79 patients were in class A (VO_2_ ≥ 11 mL/kg/min) and 46 patients were in class B (VO_2_ <11 mL/kg/min). As for post-surgical pain management, 20 and 11 patients received acetaminophen in class A and class B, respectively (*p* = 0.861). All the patients in each class received NSAIDs (regular use) and opioids (use on requirement). The mean number of using of different types of NSAIDs and opioids was 1.01 ± 0.25 and 1.58 ± 0.91 in class A, 1.00 ± 0.37 and 1.85 ± 0.84 in class B, respectively (*p* = 0.820 and 0.110, respectively). One patient in class A and One patient in class B received additional local regional analgesia *via* TPB.

The outcome comparisons according to VO_2_ at VT are shown in Table 3B. For the primary outcomes, no significant differences were found in ICU length of stay (*p* = 0.110), hospital length of stay (*p* = 0.112), ETT (p = 0.127), and CTT (*p* = 0.124) between the two classes. For the secondary outcomes, no significant differences between the two classes were observed in clinical complications (*p* = 0.942), CXR-diagnosed complications (*p* = 0.152), and clinical or CXR-diagnosed complications (*p* = 0.417).

**Table 3B:** Outcomes comparisons by oxygen consumption at anaerobic threshold (VO_2_ at VT).

			***P*-value**
**Primary outcomes**			
ICU length of stay (hour)	class A (*n =* 79)	8.53	0.110
	class B (*n =* 46)	10.19	
Hospital length of stay (day)	class A (*n =* 79)	8.37	0.112
	class B (*n =* 46)	8.00	
Endotracheal intubation time (hour)	class A (*n =* 79)	8.97	0.127
	class B (*n =* 46)	10.92	
Chest tube insertion time (hour)	class A (*n =* 78)	108.64	0.124
	class B (*n =* 46)	108.98	
**Secondary outcomes**			
Clinical complication	class A (*n =* 79)	0.59	0.942
	class B (*n =* 46)	0.59	
CXR- diagnosed complication	class A (*n =* 79)	1.03	0.152
	class B (*n =* 46)	1.33	
Clinical or CXR- diagnosed complication	class A (*n =* 79)	1.29	0.417
	class B (*n =* 46)	1.43	

*Clinical complications included 4 aspects: air (subcutaneous emphysema, pneumothorax, continuous air leakage over water-seal bottle), fluid (pleural effusion), lung (atelectasis), infection (fever, empyema); each aspect counted for 1 point. *CXR-diagnosed complication included 4 aspects: air (subcutaneous emphysema, pneumothorax, pneumomediastinum, hydropneumothorax), fluid (pleural effusion, pulmonary edema), lung (atelectasis), infection (pneumonia, empyema); each aspect counted for 1 point.

*Clinical or CXR-diagnosed complication included 4 aspects: air, fluid, lung, infection; each aspect counted for 1 point either clinical complication, CXR- diagnosed complication or both were noted.

*One participant was excluded from chest tube insertion time due to dischargement without removing chest tube.

### Comparisons of Outcomes According to V_E_/V_CO2_ Slope

On the basis of the 2016 ESC guidelines, 90, 29, and 6 patients were in class I (V_E_/V_CO2_ slope <30), class II (30 < V_E_/V_CO2_ slope <35.9), and class III (36 < V_E_/V_CO2_ slope <44.9), respectively. None of the patients belonged to class IV (V_E_/V_CO2_ slope > 45). As for post-surgical pain management, 22, 9, and 0 patients received acetaminophen in class I, class II, and class III, respectively (*p* = 0.274). All the patients in each class received NSAIDs (regular use) and opioids (use on requirement). The mean number of using of different types of NSAIDs and opioids was 1.0 ± 0.28 and 1.63 ± 0.88 in class I, 0.93 ± 0.37 and 1.93 ± 0.88 in class II, and 1.00 ± 0.00 and 1.17 ± 0.98 class III, respectively (*p* = 0.275 and 0.105, respectively). One patient in class I and one patient in class III received additional local regional analgesia *via* TPB.

The outcome comparisons according to V_E_/V_CO2_ slope are shown in Table 3C. No significant differences were found in ICU length of stay (*p* = 0.414), hospital length of stay (*p* = 0.661), ETT (*p* = 0.364), and CTT (*p* = 0.722) among classes I, II, and III. The comparisons of secondary outcomes showed no significant differences in clinical complications (*p* = 0.269), CXR-diagnosed complications (*p* = 0.896), and clinical or CXR-diagnosed complications (*p* = 0.910).

**Table 3C:** Outcomes comparisons by slope of minute ventilation and carbon dioxide production (V_E_/V_CO2_ slope).

			***P*-value**
**Primary outcomes**		
ICU length of stay (hour)	class I (*n =* 90)	8.57	0.414
	class II (*n =* 29)	11.37	
	class III (*n =* 6)	6.94	
Hospital length of stay (day)	class I (*n =* 90)	8.27	0.661
	class II (*n =* 29)	8.38	
	class III (*n =* 6)	7.00	
Endotracheal intubation time (hour)	class I (*n =* 90)	9.03	0.364
	class II (*n =* 29)	12.23	
	class III (*n =* 6)	7.28	
Chest tube insertion time (hour)	class I (*n =* 90)	110.10	0.722
	class II (*n =* 28)	108.88	
	class III (*n =* 6)	88.21	
**Secondary outcomes**		
Clinical complication	class I (*n =* 90)	0.64	0.269
	class II (*n =* 29)	0.52	
	class III (*n =* 6)	0.17	
CXR- diagnosed complication	class I (*n =* 90)	1.11	0.896
	class II (*n =* 29)	1.21	
	class III (*n =* 6)	1.17	
Clinical or CXR- diagnosed complication	class I (*n =* 90)	1.32	0.910
	class II (*n =* 29)	1.41	
	class III (*n =* 6)	1.33	

*Clinical complications included 4 aspects: air (subcutaneous emphysema, pneumothorax, continuous air leakage over water-seal bottle), fluid (pleural effusion), lung (atelectasis), infection (fever, empyema); each aspect counted for 1 point. *CXR-diagnosed complication included 4 aspects: air (subcutaneous emphysema, pneumothorax, pneumomediastinum, hydropneumothorax), fluid (pleural effusion, pulmonary edema), lung (atelectasis), infection (pneumonia, empyema); each aspect counted for 1 point. *Clinical or CXR-diagnosed complication included 4 aspects: air, fluid, lung, infection; each aspect counted for 1 point either clinical complication, CXR- diagnosed complication or both were noted. *One participant was excluded from chest tube insertion time due to dischargement without removing chest tube. *Ventilatory class IV was excluded due to insufficient samples (Ventilatory class IV, n = 0).

## Discussion

In this study, we aimed to investigate the influence of exercise testing-guided perioperative cardiopulmonary rehabilitation by applying physiotherapy interventions in the perioperative period. Our results showed that tailored perioperative cardiopulmonary rehabilitation based on CPET risk stratifications allowed patients with lung cancer with different risk levels to achieve comparable clinical and imaging outcomes after surgery. A peak VO_2_ of <10 mL/kg/min or 35% of the predicted value has been considered the threshold for prohibiting major surgeries (18). Among all variables, high values of V_E_ and V_CO2_, expressed as the V_E_/V_CO2_ slope, imply ventilatory inefficiency and have long been associated with poor outcomes in patients with chronic heart failure (19). A higher V_E_/V_CO2_ slope has also been proven to be highly correlated with respiratory complications and mortality after pulmonary resection (13). Furthermore, the ESC guidelines have established adequate criteria for the preoperative assessment of perioperative and postoperative risks and long-term prognosis. Postoperative complications can be well predicted using three CPET variables [peak VO_2_ (10, 20–27), VO_2_ at VT (28), and V_E_/V_CO2_ slope (13, 29, 30). as reported in several articles published from 2001 to 2021. Therefore, CPET is currently recommended as part of the preoperative evaluation of lung cancer patients with respiratory comorbidities and/or functional limitations (18, 31). Under current guidelines, CPET is recommended only for those patients with lower FEV1, diffusing capacity of the lung for carbon monoxide (DLCO), or their predicted postoperative (ppo) values, that is FEV1 or DLCO <80% predicted by European Respiratory Society/European Society of Thoracic Surgeons (18), or ppoFEV1 or ppoDLCO <30% by American College of Chest Physician (31). We thought that even though the spirometric values correlate strongly with the severity of lung obstruction, they can't provide direct information regarding the degree of gas exchange and cardiovascular reserve (32). On the contrary, CPET reflects interactions between pulmonary function, cardiovascular status and oxygen uptake and utilization by the peripheral tissues (12). It is probable that some lung cancer patients could undergo surgery if CPET permits it even though they once excluded from surgery based on FEV1 and FVC or DLCO results (12). CPET is not only a tool for diagnosing suspected cardiovascular and respiratory diseases or for making decision to proceed to major surgery, but also a guidance of care required postoperatively (33, 34). CPET is feasible, safe, and recommended for patients with cancer prior to a physical exercise program (35). For patients with lung cancer, though only few available studies, the physician can prescribe tailored PRCR and can also asses the effectiveness of the rehabilitation based on CPET parameters (36–38). Our team has performed CPET-guided PRCR since 2017, based on the results of this current study and the low risk of major adverse events associated with performing CPET (33), routine pre-operative CPET should be highlighted for all lung cancer patients pending lung resection if it is performed in a controlled environment with continuous monitoring, appropriate equipment and well-trained personnel, without contraindications proposed by ACSM (39).

We took advantage of the prognostic value of the CPET variables to investigate the impact of perioperative cardiopulmonary rehabilitation. According to the ESC guidelines, significant differences in prognosis and postoperative complications can be expected in patients with different risk levels. However, regardless of the risk level categories defined by peak VO_2_, VO_2_ at VT, or V_E_/V_CO2_ slope, no significant differences in outcomes were observed after perioperative cardiopulmonary rehabilitation in this study. Our results showed comparable prognosis and postoperative complications after exercise testing-guided perioperative cardiopulmonary rehabilitation in patients with different risk levels, suggesting that perioperative cardiopulmonary rehabilitation results in better prognosis and fewer postoperative complications. To clarify this phenomenon, we attempted to compare our results with those of relevant previous studies. Cavalheri et al. (40) published a meta-analysis in 2017, focusing on the effect of preoperative exercise training on postoperative outcomes in patients with NSCLC. The population in this meta-analysis was patients scheduled to undergo lung resection for NSCLC, divided into the preoperative exercise training and no exercise training groups. In terms of CTT and hospital length of stay, our study patients showed significantly superior outcomes to those of the control group (no exercise training group) and comparable outcomes to those of the experimental group (preoperative exercise training group) in the meta-analysis. The last decades have seen increasing interest in investigating the impact of the application of cardiopulmonary rehabilitation in candidates for lung resection or other surgical procedures, and studies have demonstrated that perioperative cardiopulmonary rehabilitation has beneficial effects on postoperative prognosis and complications.

Some systematic review and meta-analysis studies demonstrated that superior preoperative CPET values, especially peak VO2, were significantly associated with improved postoperative outcomes in patients undergoing cancer surgery (41). The reason for this phenomenon remains uncertain but is gradually being elucidated. As the VO_2_ level is influenced by the combined contribution of the heart, lungs, oxygen transport system, and skeletal muscles to external work, it is a comprehensive indicator of the general physical status. Meanwhile, the V_E_/V_CO2_ slope is a more specific expression of ventilatory efficiency (42). Through perioperative cardiopulmonary rehabilitation consisting of deep breathing exercises, coughing techniques, early mobilization, and progressive shoulder/thoracic mobility exercises, we provided appropriate cardiopulmonary training to our participants in the preoperative phase, resulting in improved ventilatory efficiency and physical status and reducing the individual risks of perioperative or postoperative complications. Overall, postoperative cardiopulmonary rehabilitation was performed to improve the patients' functional ability in the short term and to accelerate their physical recovery, especially cardiopulmonary function.

### Limitations

This study must be viewed in light of some limitations. First, our study was a retrospective analysis. The results on the relationship between risk levels and PPCs in patients with lung cancer should be interpreted with caution. Conclusions on the direction of the relationships cannot be drawn. Second, the patients were randomly recruited from a single medical center in Southern Taiwan. Therefore, the results may be generalizable only to similar populations, although the distributions of patients according to lung cancer type and age were similar to the data from the national survey in Taiwan. Third, the diagnosis of postoperative complications may be different and subjective depending on the doctor or radiologist. Although we collected the data of each participant as comprehensively as possible, artificial errors in progression notes, discharge notes, or nursing records might exist. Forth, all the patients with lung cancer in this study presented with relatively normal FEV1, regardless of absolute or measured to predicted values. This finding was not surprising given that patients are suitable for lobectomy if FEV1 is >1.5L according to current guideline. However, given that our team has performed CPET-guided PRCR since 2017 with well-equipment, well-experienced physiatrist and allied-health staffs, all the patients in this study received pre-operative CPET which was against current guideline that CPET is recommended for those with either FEV1 or DLCO <80% predicted. Our results in this study should be interpreted carefully in different clinical settings.

## Conclusion

In our study, patients with lung cancer undergoing VATS with different risk levels showed comparable prognosis and postoperative complications after undergoing exercise testing-guided perioperative cardiopulmonary rehabilitation. We highly recommend performing preoperative CPET if it could be performed in a controlled environment with continuous monitoring, appropriate equipment, and well-trained personnel, and starting perioperative cardiopulmonary rehabilitation as soon as possible in patients undergoing surgery for lung cancer. Future larger and randomized control studies are warranted to confirm the clinical effectiveness of exercise testing-guided perioperative cardiopulmonary rehabilitation.

## Data Availability Statement

The original contributions presented in the study are included in the article/supplementary material, further inquiries can be directed to the corresponding authors.

## Ethics Statement

This study was approved by the Institutional Review Board of Kaohsiung Veterans General Hospital (Number: VGHKS17-CT11-11). The patients/participants provided their written informed consent to participate in this study.

## Author Contributions

Conceptualization: S-HT and K-LL. Data curation: W-HC, E-KT, and J-HC. Methodology: W-HC and Y-JT. Resources: K-LL. Supervision: E-KT and K-LL. Writing—original draft: W-HC and S-HT. Writing—review and editing: Y-JT and G-BC. All authors contributed to the article and approved the submitted version.

## Funding

This study was supported by the grant of Ministry of Science and Technology of Taiwan (R.O.C.), Grant Number: MOST 110-2314-B075B-005-MY2.

## Conflict of Interest

The authors declare that the research was conducted in the absence of any commercial or financial relationships that could be construed as a potential conflict of interest.

## Publisher's Note

All claims expressed in this article are solely those of the authors and do not necessarily represent those of their affiliated organizations, or those of the publisher, the editors and the reviewers. Any product that may be evaluated in this article, or claim that may be made by its manufacturer, is not guaranteed or endorsed by the publisher.

## Abbreviations

NSCLC: non-small cell lung cancer
ESC: European Society of Cardiology
CPET: cardiopulmonary exercise testing
Peak VO_2_: peak oxygen consumption
VO_2_ at VT: VO_2_ at the ventilatory threshold
V_E_/V_CO2_ slope: slope of minute ventilation to carbon dioxide production
PPC: postoperative pulmonary complication
VATS: video-assisted thoracic surgery
IMT: inspiratory muscle training
ICU: intensive care unit
ETT: endotracheal intubation time
CTT: chest tube insertion time
CXR: plain chest radiography
BMI: body mass index
FVC: forced vital capacity
FEV1: forced expiratory volume in 1 second
MVV: maximum voluntary ventilation.
